# Retrospective comparison of direct in-bore magnetic resonance imaging (MRI)-guided biopsy and fusion-guided biopsy in patients with MRI lesions which are likely or highly likely to be clinically significant prostate cancer

**DOI:** 10.1007/s00345-017-2085-6

**Published:** 2017-09-04

**Authors:** Wulphert Venderink, Marloes van der Leest, Annemarijke van Luijtelaar, Wendy J. M. van de Ven, Jurgen J. Fütterer, J. P. Michiel Sedelaar, Henkjan J. Huisman

**Affiliations:** 1 Department of Radiology and Nuclear Medicine, Radboud University Medical Center, P.O.Box 9101, 6500 HB Nijmegen, The Netherlands; 2 Department of Urology, Radboud University Medical Center, Nijmegen, The Netherlands

**Keywords:** MRI-TRUS fusion, Direct in-bore, Prostate, Biopsy, Detection rate, PI-RADS

## Abstract

**Purpose:**

To compare clinically significant prostate cancer (csPCa) detection rates between magnetic resonance imaging (MRI)–transrectal ultrasound (TRUS) fusion-guided prostate biopsy (FGB) and direct in-bore MRI-guided biopsy (MRGB).

**Methods:**

We performed a comparison of csPCa detection rates between FGB and MRGB. Included patients had (1) at least one prior negative TRUS biopsy; (2) a Prostate Imaging Reporting and Data System (PI-RADS) 4 or 5 lesion and (3) a lesion size of ≥8 mm measured in at least one direction. We considered a Gleason score ≥7 being csPCa. Descriptive statistics with 95% confidence intervals (CI) were used to determine any differences.

**Results:**

We included 51 patients with FGB (59 PI-RADS 4 and 41% PI-RADS 5) and 227 patients with MRGB (34 PI-RADS 4 and 66% PI-RADS 5). Included patients had a median age of 69 years (IQR, 65–72) and a median PSA level of 11.0 ng/ml (IQR, 7.4–15.1) and a median age of 67 years (IQR, 61–70), the median PSA 12.8 ng/ml (IQR, 9.1–19.0) within the FGB and the MRGB group, respectively. Detection rates of csPCA did not differ significantly between FGB and MRGB, 49 vs. 61%, respectively.

**Conclusion:**

We did not detect significant differences between FGB and MRGB in the detection of csPCa. The differences in detection ratios between both biopsy techniques are narrow with an increasing lesion size. This study warrants further studies to optimize selection of best biopsy modality.

## Introduction

Several major changes have taken place in the last decade regarding the diagnosis of prostate cancer (PCa). Most important is the introduction of multiparametric magnetic resonance imaging (mpMRI). mpMRI allowed for an accurate detection and localization of clinically significant (cs)PCa. It also made it possible to perform targeted biopsy instead of 10–12 core systematic transrectal ultrasound (TRUS)-guided biopsy and it thus enables to reduce the number of biopsy needles [1, 2]. Nowadays, mpMRI is recommended by the European Association of Urology (EAU) in men with a persistent clinical suspicion of PCa despite a negative TRUS biopsy [3]. These recommendations are made because TRUS biopsy is under-diagnosing csPCa, especially in lesions anteriorly located in the prostate [4].

After mpMRI, suspicious areas can be targeted using the obtained information. Targeted biopsy can be done, for example, direct in-bore MR-guided (MRGB). MRGB is able to accurately target suspicious lesions; however, the procedure is time-consuming, expensive and in most countries very limited accessible [5, 6]. For these reasons, MRI-TRUS fusion-guided biopsy (FGB) is more commonly performed. In FGB, previously obtained mpMRI information is fused (cognitively or software assisted) with real-time TRUS images. MR “slot-time” can be saved because the biopsy can be performed ultrasound-guided rather than MR-guided. This enables the procedure to be less expensive and much more readily available. Also, FGB allows a urologist to perform a 10–12 core systematic TRUS biopsy in addition to targeted biopsy as systematic TRUS biopsy still detects csPCa in up to 10% of patients which would be missed in a targeted-only approach [7–9]. In our institution, only patients with lesions larger than 8 mm measured in at least one direction are considered eligible for FGB as we expected FGB in those lesions to be as accurate as MRGB [10, 11].

With FGB and MRGB increasingly being practiced, there is a need to determine whether those techniques yield comparable csPCa detection rates. Nowadays, as far as we know, only one study was performed which compared the two targeted biopsy approaches [12]. Therefore, the aim of our study is to compare the difference in the detection of csPCa between both biopsy procedures.

## Methods

### Patients

In our institution, 82 patients had FGB between December 2014 and December 2016. Of these patients, 51 met the next inclusion criteria: (1) at least one prior negative TRUS biopsy; (2) a Prostate Imaging Reporting and Data System (PI-RADS) 4 or 5 lesion localized on prior mpMRI performed in our institution and (3) a lesion size of ≥8 mm measured in at least one direction. To compare, we searched our institutional MRGB database, starting from January 2012. This reference database contained 227 patients with the same inclusion criteria. The study was approved by our institutional review board.

### mpMRI

mpMRI was performed on a 3.0 T MR-scanner (Siemens, Skyra) with a pelvic phased-array coil. Tri-planar anatomical T2-weighted images (T2W), axial dynamic contrast-enhanced images (DCE) and axial diffusion-weighted (DW) images were obtained. Images were analyzed and reported according to PI-RADS version 1 or 2 by six radiologists with varying experience in prostate MR reading (2–20 years) [13, 14].

### Software-assisted registration

Prior to the FGB procedure, Digital Imaging and Communications in Medicine (DICOM) images were uploaded to the ultrasound device (Aplio 500, Toshiba Medical Systems). An electromagnetically (EM) tracking field generator was placed near the pelvis of the patient and an EM tracking sensor was attached to the free-hand operated transrectal ultrasound probe (PVT-781 VT) so that real-time movement tracking is allowed. Uploaded axial T2 W images and ultrasound images were displayed side by side. Rigid image registration was acquired by selecting landmarks visible on both the ultrasound images and the uploaded T2W images. A landmark (e.g., cysts, verumontanum or BPH nodules) as close as possible to the suspicious lesion was chosen to enable the most reliable registration. After software-assisted registration, we cognitively enhanced the fusion as rigid image registration is often distorted by the deformation of the prostate caused by the introduction of the ultrasound probe for example. We only performed targeted biopsy without additional 10–12 core random biopsy. Procedure time was typically 10–20 min. The procedure was performed without using anesthetics. The described registration method does not allow for a confirmation of the needle position in the prostate.

As our institution was much more experienced in MRGB at the time of the introduction of FGB in our hospital, we offered FGB only to patients with a PI-RADS 4 or 5 lesion and a lesion size of ≥8 mm measured in at least one direction. Patients with smaller lesions and lesions scored PI-RADS 3 were immediately offered MRGB. Patients who preferred MRGB over FGB were offered MRGB and vice versa. FGB was performed by one radiologist without prior prostate biopsy experience.

### Direct in-bore MR-guided biopsy

During MRGB, patients are positioned in a prone position. A needle guide is rectally inserted. Prior to biopsy, additional axial T2W and axial DW images were made to confirm the localization of the lesion. True fast imaging with steady-state free precession (TRUFI) images were used to direct the manually adjustable needle guide. After each biopsy, the position of the needle was confirmed with TRUFI images. The accuracy of the needle position was assessed by one of the prostate MR experienced radiologists. No anesthetics were used during this procedure. All biopsies were performed transrectal without adding 10–12 core systematic TRUS biopsies. Procedure time is typically 45–60 min.

### Histopathology

All biopsy cores were evaluated by one of the three dedicated uropathologist. Pathologists were not blinded for the biopsy method or the mpMRI findings. We considered a Gleason score ≥7 being clinically significant. In case a patient does have multiple lesions, we used the index lesion (according to PI-RADS) for the analysis. In case a patient had a lesion next to the index lesion which did not match the inclusion criteria, we did biopsy the lesion; however, we did not evaluate the results in this study.

### Statistical analysis

We used descriptive statistics with 95% confidence intervals (CI) with a continuity correction factor to calculate potential differences between the two techniques. Additionally, we used Chi-squared statistics to calculate for significant differences between cohorts. A *p* value ≤0.05 was considered statistically significant. Analyses were conducted using IBM SPSS Statistics (Version 22).

## Results

### Patient characteristics

The 51 included patients having FGB had 58 lesions to target. Those patients had a median age of 69 years (IQR, 65–72) and a median PSA level of 11.0 ng/ml (IQR, 7.4–15.1). They had a median of 2 (IQR, 1–2) previous negative TRUS biopsy sessions and had a median PSA density 0.18 ng/ml/ml (IQR, 0.1–0.3). Overall, 58.8% (30/51) were biopsied because of a PI-RADS 4 index lesion and 41.2% (21/50) because of PI-RADS 5.

The 227 patients in the reference database having MRGB had 261 biopsied lesions. The median age was 67 years (IQR, 61–70), the median PSA 12.8 ng/ml (IQR, 9.1–19.0) and the median PSA density ng/ml/ml 0.23 (IQR, 0.15–0.40). Patients had a median of 2 (IQR, 1–3) prior negative TRUS biopsy sessions. Overall, 33.9% (77/227) were biopsied because of a PI-RADS 4 index lesion and 66.1% (150/227) because of PI-RADS 5. Further patient and lesion characteristics are specified in Table 1.

**Table 1:** Patient and lesion characteristics

Patient characteristics	FGB (*n* = 51)	MRGB (*n* = 227)
Age, years, median (IQR)	69 (65–72)	67 (61–70)
PSA level, ng/ml, median (IQR)	11.0 (7.4–15.1)	12.8 (9.1–19)
Prostate volume, ml, median (IQR)	63.0 (46-86.0)	53.0 (36.5–78.0)
PSA density, ng/ml/ml, medain (IQR)	0.18 (0.1–0.3)	0.23 (0.15–0.4)
PI-RADS score index lesion, *n* (%)		
4	30 (58.8)	77 (33.9)
5	21 (41.2)	150 (66.1)
Time between mpMRI and biopsy, days, median (IQR)	28 (21–43)	29 (17–42)
No. of prior TRUS biopsies, median (IQR)	2 (1–2)	2 (1–3)

Lesion characteristics	FGB (*n* = 58)	MRGB (*n* = 261)
PI-RADS score, *n* (%)		
4	37 (63.8)	101 (38.7)
5	21 (36.2)	160 (61.3)
Biopsies per lesion, n, median (IQR)	3 (2–3)	2 (2–3)

*FGB* fusion-guided biopsy, *MRGB* direct in-bore magnetic resonance-guided biopsy, *yr* year, *IQR* Inter quartile range; PSA = prostate specific antigen; *PI-RADS* Prostate Imaging Reporting and Data System; mpMRI = multiparametric magnetic resonance imaging; TRUS = transrectal ultrasound-guided biopsy

### Prostate cancer detection

In patients having FGB, csPCa was detected in 49.0% (25/51) and any PCa in 66.7% (34/51). The csPCa detection rate in patients with PI-RADS 4 or 5 was 33.3 (10/30) and 71.4% (15/21), respectively. In these subcohorts, any PCa was detected in 56.7 (17/30) and 81.0% (17/21), respectively.

The detection rates in patients having MRGB were 61.2% (139/227) for csPCa and 85.0% (193/227) for any PCa. This is a difference in favor of MRGB of 12.2 (*p* = 0.11) and 18.3 (*p* < 0.05) percentage points, respectively.

The csPCa detection rates favored MRGB in patients with a lesion scored PI-RADS 4 with 16.0 (*p* = 0.13) percentage points. In patients with PI-RADS 5, FGB reached a csPCa detection rate which was 4.1 (*p* = 0.71) percentage points higher than that of MRGB (Table 2).

**Table 2:** Detection rates of (cs)PCa

Detection rates	FGB (*n* = 51)	MRGB (*n* = 227)	Difference^a^, (95% CI)	*p* values^b^
Overall				
Any PCa, % (*n*)	66.7 (34)	85.0 (193)	18.3 (5.0–33.7)	<0.05
csPCa, % (*n*)	49.0 (25)	61.2 (139)	12.2 (−3.5–27.6)	0.11
PI-RADS 4	*n* = 30	*n* = 77		
Any PCa, % (*n*)	56.7 (17)	72.7 (56)	16.1 (−4.8–37.2)	0.11
csPCa, % (*n*)	33.3 (10)	49.4 (38)	16.0 (−6.6–35.3)	0.13
PI-RADS 5	*n* = 21	*n* = 150		
Any PCa, % (*n*)	81.0 (17)	91.3 (137)	10.4 (−3.7–34.2)	0.14
csPCa, % (*n*)	71.4 (15)	67.3 (101)	4.1 (−20.7–22.4)	0.71

*FGB* fusion-guided biopsy, *MRGB* direct in-bore magnetic resonance-guided biopsy, *PCa* prostate cancer, *cs* clinically significant (Gleason score ≥ 7), *PI-RADS* Prostate Imaging Reporting and Data System

^a^Differences are shown in percentage points

^b^ *p* values are calculated using Chi-squared statistics

**Fig. 1:**
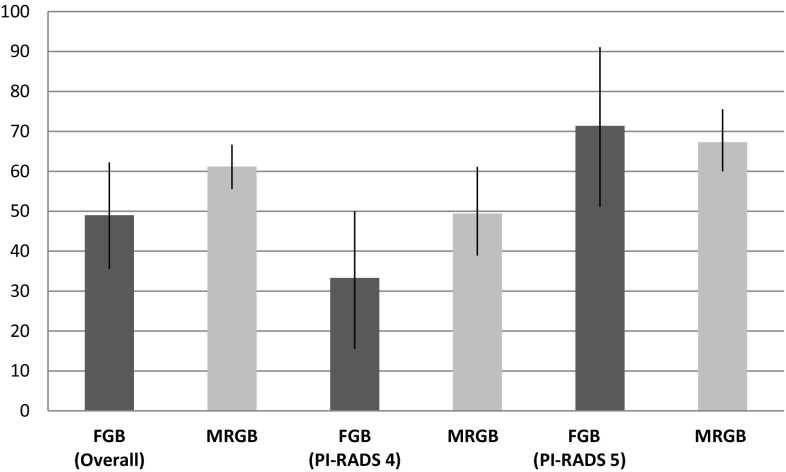
CsPCa detection of FGB and MRGB. CsPCa detection rates of FGB and MRGB displayed overall and per PI-RADS classification. The *bar chart* represents the detection rates and the *black lines* indicate the 95% confidence intervals. *csPCa* clinically significant prostate cancer (Gleason score ≥7), *FGB* fusion-guided biopsy; direct in-bore magnetic resonance imaging-guided biopsy, *PI*-*RADS* Prostate Imaging Reporting and Data System

**Fig. 2:**
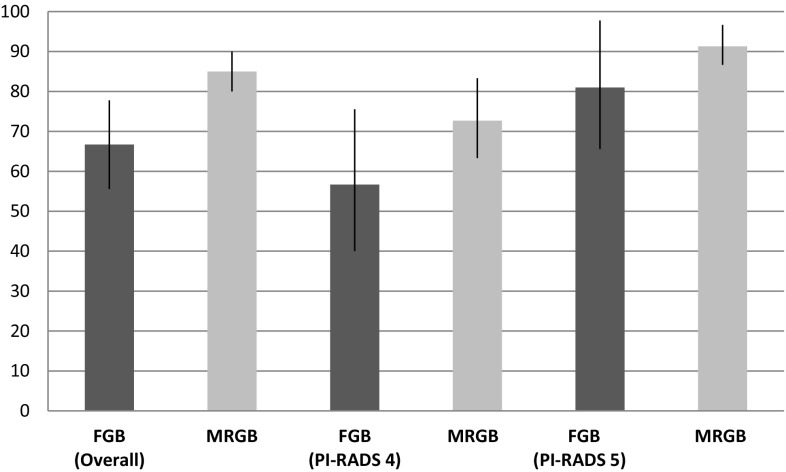
Any PCa detection of FGB and MRGB. PCa detection rates of FGB and MRGB displayed overall and per PI-RADS classification. The *bar charts* represent the detection rates and the *black lines* indicate the 95% confidence intervals. *PCa* prostate cancer, *FGB* fusion-guided biopsy, *MRGB* direct in-bore magnetic resonance imaging-guided biopsy, *PI*-*RADS* Prostate Imaging Reporting and Data System

Figures 1 and 2 represent the csPCa and any PCa detection rates, respectively, per (sub)cohort of both techniques with 95% confidence intervals.

### csPCa detection correlated to lesion size

**Fig. 3:**
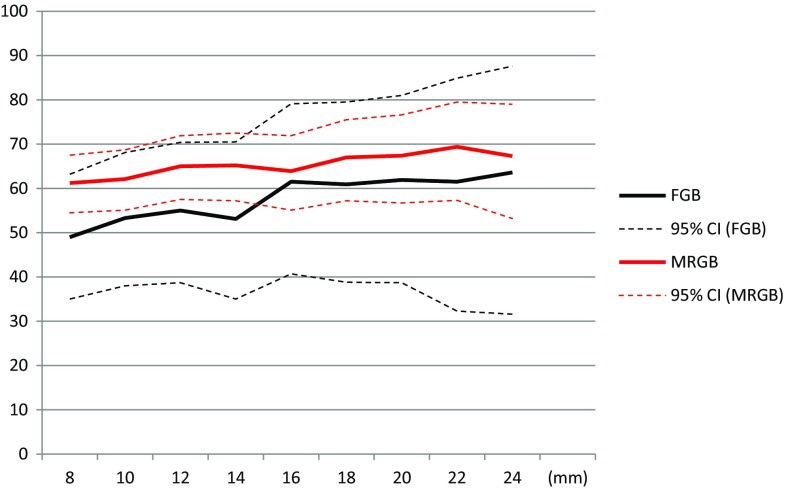
csPCa detection with different lesion sizes. csPCa detection rates and 95% CI for both FGB and MRGB with different lesion sizes in millimeters. The *dotted lines* represent the 95% CI. *csPCa* clinically significant prostate cancer, *CI* confidence interval, *FGB* fusion-guided biposy, *MRGB* direct in-bore magnetic resonance imaging-guided biopsy, *mm* millimeter

Applying a minimal lesion size of 16 mm instead of 8 mm increases the csPCa detection rate from 49 (95% CI, 35.0–63.2) to 61.5% (95% CI, 40.7–79.1) for FGB and from 61.2 (95% CI, 54.5–67.5) to 63.9% (95% CI, 55.1–71.9) for MRGB. Further increasing the minimal lesion size to 24 mm results in a csPCa detection ratio of 63.6 (95% CI, 31.6–87.6) and 67.3% (95% CI, 53.2–79.0) for FGB and MRGB, respectively. In Fig. 3, the detection ratios for csPCa are displayed correlated with the minimal lesion size.

**Fig. 4:**
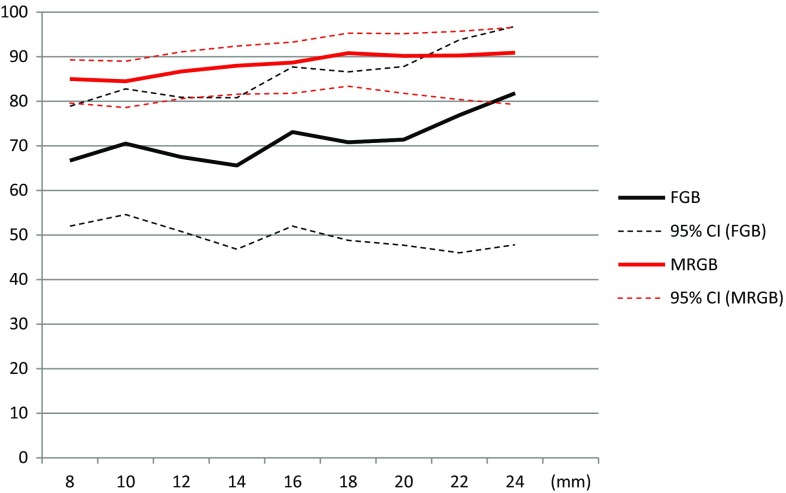
Any PCa detection with different lesion sizes. Any PCa detection rates and 95% CI for both FGB and MRGB with different lesion sizes in millimeters. The *dotted lines* represent the 95% CI. *PCa* prostate cancer, *CI* confidence interval, *FGB* fusion-guided biposy, *MRGB* direct in-bore magnetic resonance imaging-guided biopsy, *mm* millimeter

The detection rate of any PCa for FGB increases from 66.7 (95% CI, 52.0–78.9) with a minimal lesion size of 8 mm to 73.1% (95% CI, 52.0–87.7) and 81.8% (95% CI, 47.8–96.8) in case lesions of 16 mm or 24 mm would have been biopsied, respectively. Detection rates of any PCa for MRGB would increase from 85.0 (95% CI, 79.6–89.3) to 88.7% (95% CI, 81.8–93.3) and 90.9% (95% CI, 79.3–96.6) applying a minimal biopsy threshold of 16 and 24 mm, respectively. In Fig. 4, we supplied the detection rates of any PCa with 95% CI with different minimal lesion sizes.

### Follow-up after negative fusion-guided biopsy

Within the cohort of 17 patients with a negative biopsy outcome after FGB, two patients had a follow up (FU) MRGB performed within 2 months. In both patients, PCa was detected: in one a GS 3 + 3 and in one a GS 2 + 3. In one patient, a radiologist downgraded the level of suspicion from PI-RADS 5 to PI-RADS 2 after another mpMRI was performed a year after FGB. In another five patients, PSA decreased and, therefore, no follow-up mpMRI was performed. In the remaining nine patients, follow-up is unknown.

## Discussion

This study evaluated the performance of FGB compared to MRGB. We did not detect statistically significant differences between FGB and MRGB for csPCa in patients with lesions scored PI-RADS 4 or 5 with a minimal lesion size of 8 mm measured in at least one direction (49 vs 61%). We neither detected statistically significant differences when evaluating the results for PI-RADS 4 and 5 separately.

As far as we know, at the moment, only Arsov et al. [12]. performed a study in which two MR-targeted prostate biopsy approaches were compared. They compared PCa detection rates between an MRGB approach alone and an FGB approach combined with systematic TRUS biopsy. As interim analyses did not identify an important improvement in detection rates for the combined approach, the study was halted. Evaluating their results, exclusively comparing the two targeted biopsy approaches, in-bore biopsy reaches a csPCa detection rate of 29% (31/106) compared to a detection rate of 26% (27/104) in patients having FGB.

As in Arsov et al., we did not detect significant differences in csPCa detection rates. Remarkably, the csPCa detection rates in our study are higher than those of Arsov et al. this could be well explained by the use of different inclusion criteria in both studies. We only included patients with lesions scored PI-RADS 4 or 5 and a minimal lesion size of 8 mm. Therefore, our results cannot be reliably compared with their results.

Comparing the cohorts in our study for PI-RADS 4 and 5 separately, we demonstrated a slightly lower csPCa detection rate in PI-RADS 4 lesions for FGB, although this result was not statistically significant. On the other hand, in patients with a PI-RADS 5 lesion, the detection rate of csPCa is slightly higher in the cohort having FGB; again, this was not a statistically significant difference. The higher detection rate for FGB in lesions score PI-RADS 5 is probably caused by the fact that a PI-RADS 5 lesion is most commonly larger than a lesion scored PI-RADS 4. With both FGB and MRGB experience, we noticed that larger lesions and lesions with a PI-RADS score of 5 are often visible on ultrasound during FGB, which allowed us to target such lesions accurately. This is supported by the results of our evaluation of detection rates correlated to the lesion size as displayed in Figs. 3, 4. The difference in detection rates between the two biopsy techniques is narrow when applying a higher threshold for the minimal lesion size. These results, however, should be evaluated with caution. The sample size is getting smaller when increasing the threshold of the lesion size and, as a consequence, the 95% CI is widening. However, in our institution, both FGB and MRGB are being practiced. As we used a fusion platform based on rigid image registration, a cognitive enhancement is required to biopsy a suspicious lesion reliably. This enhancement is most reliable in case a lesion appears to be visible on gray scale ultrasound after the software-assisted rigid image registration is completed. We observed that most cases required a cognitive enhancement. Though, in most cases cognitive enhancement was possible as lesions often appear to be visible on grayscale ultrasound after the software-assisted image registration was performed, especially the lesions scored PI-RADS 5 and the lesions with a larger diameter. This suggestion is supported by the presented data as the detection rate of csPCa in lesions scored PI-RADS 5 is almost equal between both biopsy techniques. The differences in detection rates between both biopsy techniques becomes smaller when applying a higher threshold of lesions sizes from where to biopsy. A further increase in ultrasound visibility of lesions may be reached by adding other ultrasound modalities like power Doppler or elastography; this may increase the diagnostic accuracy of FGB based on a rigid image registration system [15, 16].

At the moment, the studies investigating csPCa detection rates between different MR-targeted biopsy approaches did not detect statistically significant differences. In our study, this may be explained by the use of a retrospective study design with a relatively small sample size, which is an important limitation of our study. A prospective trial should be performed to investigate potential relevant differences between both biopsy techniques. Unfortunately, the prospective trial of Arsov et al. [12], which tried to address this issue, was halted and thus did not reach their required sample size. Of course, the question arises which differences in detection rates are allowed as FGB is less expensive compared to MRGB and it thus may be less accurate. To assess the required diagnostic accuracy of FGB, Health Technology Assessment studies could be helpful.

A limitation of our study is that we performed a single centre study. As a consequence, we performed FGB and MRGB on one type of machine, while nowadays, several commercially available platforms are used worldwide. In the future, a multicentre study could solve this limitation.

In our institution, only patients with lesions larger than 8 mm measured in at least one direction are considered eligible for FGB as we beforehand expected FGB to be slightly less accurate than MRGB. Thus, the results of this study do not cover lesions which are quite small. This raises the question whether FGB is an appropriate technique to target such small lesions. Unfortunately, our data are not appropriate to address this question.

Another limitation of our study is the different number of included patients in both cohorts making comparisons difficult. It would have been desirable to increase the FGB cohort, for instance to eliminate the learning curve we had. To maximize the MRGB cohort, we used a longer inclusion for that cohort. It is clear that our institution is much more experienced in MRGB than in FGB which may introduce a bias in favor of MRGB. Unfortunately, FGB was introduced in our institution at a later time.

A last limitation of our study is the distribution of PI-RADS 4 and 5 in both subgroups. The FGB cohort consists of approximately 40% of patients with a PI-RADS 5 lesion while this is almost 70% in the MRGB cohort. This is likely to influence the results in favor of MRGB.

Our findings support our persuasion of FGB having an important role in the diagnosis of csPCa in patients with suspicious lesions seen on mpMRI, especially in larger lesions. Compared to MRGB, FGB is relatively a simple technique to implement in urologist’s practice. Procedure time for example is considerably shorter for FGB. Further, in most countries MR “slot-time” is expensive and very limited available making FGB a less expensive and thus a more attractive procedure. Further, FGB allows you to perform 10–12 core systematic TRUS biopsy next to targeted biopsy which may be important as several studies are reporting up to 10% of detected csPCa with systematic biopsy which would be missed in a targeted-only approach [7, 8]. MRGB appears to be a method reserved for the institutions that are in the position to use MR “slot-time” for this procedure.

## Conclusion

We did not detect significant differences between FGB and MRGB in the detection of csPCa. The differences in detection ratios between both biopsy techniques are narrow with an increasing lesion size. This study warrants further studies to optimize selection of best biopsy modality.

## References

[1] Kirkham AP, Emberton M, Allen C (2006). How good is MRI at detecting and characterising cancer within the prostate?. Eur Urol.

[2] Sciarra A, Barentsz J, Bjartell A, Eastham J, Hricak H, Panebianco V, Witjes JA (2011). Advances in magnetic resonance imaging: how they are changing the management of prostate cancer. Eur Urol.

[3] Heidenreich A, Bastian PJ, Bellmunt J, Bolla M, Joniau S, van der Kwast T, Mason M, Matveev V, Wiegel T, Zattoni F, Mottet N, European Association of U (2014). EAU guidelines on prostate cancer. Part 1: screening, diagnosis, and local treatment with curative intent-update 2013. Eur Urol.

[4] Schouten MG, van der Leest M, Pokorny M, Hoogenboom M, Barentsz JO, Thompson LC, Futterer JJ (2017). Why and where do we miss significant prostate cancer with multi-parametric magnetic resonance imaging followed by magnetic resonance-guided and transrectal ultrasound-guided biopsy in biopsy-naive men?. Eur Urol.

[5] Felker ER, Lee-Felker SA, Feller J, Margolis DJ, Lu DS, Princenthal R, May S, Cohen M, Huang J, Yoshida J, Greenwood B, Kim HJ, Raman SS (2016). In-bore magnetic resonance-guided transrectal biopsy for the detection of clinically significant prostate cancer. Abdom Radiol (New York).

[6] Pokorny MR, de Rooij M, Duncan E, Schroder FH, Parkinson R, Barentsz JO, Thompson LC (2014). Prospective study of diagnostic accuracy comparing prostate cancer detection by transrectal ultrasound-guided biopsy versus magnetic resonance (MR) imaging with subsequent MR-guided biopsy in men without previous prostate biopsies. Eur Urol.

[7] Siddiqui MM, Rais-Bahrami S, Turkbey B, George AK, Rothwax J, Shakir N, Okoro C, Raskolnikov D, Parnes HL, Linehan WM, Merino MJ, Simon RM, Choyke PL, Wood BJ, Pinto PA (2015). Comparison of MR/ultrasound fusion-guided biopsy with ultrasound-guided biopsy for the diagnosis of prostate cancer. JAMA.

[8] Abd-Alazeez M, Kirkham A, Ahmed HU, Arya M, Anastasiadis E, Charman SC, Freeman A, Emberton M (2014). Performance of multiparametric MRI in men at risk of prostate cancer before the first biopsy: a paired validating cohort study using template prostate mapping biopsies as the reference standard. Prostate Cancer Prost Dis.

[9] Futterer JJ, Briganti A, De Visschere P, Emberton M, Giannarini G, Kirkham A, Taneja SS, Thoeny H, Villeirs G, Villers A (2015). Can clinically significant prostate cancer be detected with multiparametric magnetic resonance imaging? A systematic review of the literature. Eur Urol.

[10] van de Ven WJ, Hu Y, Barentsz JO, Karssemeijer N, Barratt D, Huisman HJ (2015). Biomechanical modeling constrained surface-based image registration for prostate MR guided TRUS biopsy. Med Phys.

[11] van de Ven WJ, Sedelaar JP, van der Leest MM, Hulsbergen-van de Kaa CA, Barentsz JO, Futterer JJ, Huisman HJ (2016). Visibility of prostate cancer on transrectal ultrasound during fusion with multiparametric magnetic resonance imaging for biopsy. Clin Imaging.

[12] Arsov C, Rabenalt R, Blondin D, Quentin M, Hiester A, Godehardt E, Gabbert HE, Becker N, Antoch G, Albers P, Schimmoller L (2015). Prospective randomized trial comparing magnetic resonance imaging (MRI)-guided in-bore biopsy to MRI-ultrasound fusion and transrectal ultrasound-guided prostate biopsy in patients with prior negative biopsies. Eur Urol.

[13] Weinreb JC, Barentsz JO, Choyke PL, Cornud F, Haider MA, Macura KJ, Margolis D, Schnall MD, Shtern F, Tempany CM, Thoeny HC, Verma S (2016). PI-RADS prostate imaging—reporting and data system: 2015, Version 2. Eur Urol.

[14] Barentsz JO, Richenberg J, Clements R, Choyke P, Verma S, Villeirs G, Rouviere O, Logager V, Futterer JJ, European Society of Urogenital R (2012). ESUR prostate MR guidelines 2012. Eur Radiol.

[15] Maxeiner A, Stephan C, Durmus T, Slowinski T, Cash H, Fischer T (2015). Added value of multiparametric ultrasonography in magnetic resonance imaging and ultrasonography fusion-guided biopsy of the prostate in patients with suspicion for prostate cancer. Urology.

[16] Postema A, Mischi M, de la Rosette J, Wijkstra H (2015). Multiparametric ultrasound in the detection of prostate cancer: a systematic review. World J Urol.

